# The Effect of Outcome vs. Process Accountability-Focus on Performance: A Meta-Analysis

**DOI:** 10.3389/fpsyg.2022.795117

**Published:** 2022-04-27

**Authors:** Ira Sharon, Anat Drach-Zahavy, Einav Srulovici

**Affiliations:** Faculty of Social Welfare and Health Sciences, Department of Nursing, University of Haifa, Haifa, Israel

**Keywords:** accountability focus, outcome accountability, process accountability, meta-analysis, nursing, performance

## Abstract

**Background:**

The foundation of a safe practice is accountability, especially outcome- rather than process-focused accountability, particularly during pandemics such as COVID-19. Accountability is an essential behavior that promotes congruence between nursing actions and standards associated with quality of care. Moreover, the scant research examining whether one accountability focus is superior in motivating humans to better task performance yields inconclusive results.

**Aims:**

Systematically examine the effect of an outcome- vs. process-accountability focus on performance and identify any moderating variables.

**Design:**

Systematic review and meta-analysis.

**Data sources:**

PsycINFO, Medline, PubMed, Scopus, and CINAHL databases, with all publications to November 2020.

**Review methods:**

A systematic search using Systematic Reviews and Meta-Analyses (PRISMA) guidelines was performed. Statistical analysis and forest plots were performed using MetaXL 5.3. Heterogeneity was presented using I^2^ statistics and Q tests, and possible publication bias was assessed with a Doi plot and the LFK index.

**Results:**

Seven studies representing nine experiments involving 1,080 participants were included. The pooled effect of the nine experiments on task performance failed to show significant differences (mean = −0.09; 95% Confidence Interval [95%CI]: −0.21, 0.03), but a significant moderating effect of task complexity was demonstrated. Specifically, outcome accountability exerts a beneficial effect in complex tasks (mean = −0.48 [95%CI: −0.62, −0.33]) whereas process accountability improves the performance in simpler tasks (mean = 0.96 [95%CI: 0.72, 1.20]).

**Conclusion:**

These findings demonstrated that accountability focus by itself cannot serve as a sole motivator of better performance, because task complexity moderates the link between accountability focus and task performance. Outcome accountability exerts a beneficial effect for more-complex tasks, whereas process accountability improves the performance of simpler tasks. These findings are crucial in nursing, where it is typically assumed that a focus on outcomes is more important than a focus on processes.

## Introduction

Accountability is considered a common, important, and complex concept, serving as a cornerstone of all societies and all the professional organizations that make up human society (Mansouri and Rowney, 2014; Hall et al., 2017). In nursing, accountability underpins safe practice and is an essential behavior that promotes congruence between nursing actions and standards associated with quality of care (Drach-Zahavy et al., 2018; Stievano and Tschudin, 2019; Buheji and Buhaid, 2020; Chen et al., 2020; Combrinck et al., 2021). However, the notion of accountability has begun to gain traction in nursing education and curricula worldwide only in recent years (Charania et al., 2017). Accountability becomes even more vital when nurses are at the forefront of fighting pandemics, such as COVID-19, as they are increasingly working independently with less supervision from managers. During the early stages of the pandemic, when little was known about the new pandemic and how to fight it, nurses showed professionalism, perseverance, and commitment to their patients and the healthcare system (Li and Luo, 2020). Moreover, nurses showed a high incidence of COVID infection and even death (Jackson et al., 2020). Management is thus forced, now more than ever, to rely on the moral code of nurses, as expressed by their sense of accountability. Additionally, accountability helps nurses cope with the emotional, physical, and informational strain caused by their work environment during routine times as well as during crises (Turale et al., 2020). Accountability is defined as a complex, three-dimensional concept: (a) the individual takes responsibility for their actions (*responsibility*); (b) the individual agrees that their decisions or actions will be assessed by a meaningful audience (*transparency*); and (c) the individual acknowledges that rewards or sanctions will be imposed in accordance with this assessment (*answerability*) (Srulovici and Drach-Zahavy, 2017; Drach-Zahavy et al., 2018).

The research of accountability has grown considerably during the last decades, but so far, the main conclusion is that the impacts of accountability are inconclusive (Hall et al., 2017). In the quest to make sense of the disparate findings, researchers (Chang et al., 2013; Hall et al., 2017; Patil et al., 2017) have refined the concept of accountability and differentiated between outcome and process accountability (accountability focus). Yet, whether the inconclusive findings could be attributed to this distinction needs further research.

Specifically in nursing, higher accountability (as a personal characteristic) was associated with nurses’ improved performance and lower frequency of missed nursing care (Srulovici and Drach-Zahavy, 2017; Drach-Zahavy et al., 2018; Drach-Zahavy and Srulovici, 2019). Nurses who failed to be accountable have provided lower quality and safe cares, leading to prolonged patients’ recovery periods, or even patients’ deterioration (Srulovici and Drach-Zahavy, 2017). Yet, when personal accountability was not accompanied by organizational accountability, nurses felt more strain and were more inclined to quit their job (Leonenko and Drach-Zahavy, 2016; Drach-Zahavy and Leonenko, 2019).

Given that accountability make opposite predictions about its outcomes (de Langhe et al., 2011; Chang et al., 2017; Patil et al., 2017), research revolves around strategies for reconciling these opposites. Two partly overlapping approaches have been suggested: (a) differentiating between types of accountability focus, namely process vs. outcome accountability; or (b) contingency approaches that propose that the effects of accountability are contingent on moderating variables, such as task type (De Dreu et al., 2006; Scholten et al., 2007).

Only scant research in and out of the nursing literature has differentiated outcome accountability from process accountability. Whereas outcome accountability is the expectation of individuals to be accountable for the final product of their decision without considering the process by which the decision was made, process accountability is the expectation of individuals to be judged for their decision-making course, regardless of the outcome of the decision (Hall et al., 2017). In nursing, outcome accountability is prevalent, for example, when nurses are judged according to their attainment of quality measures (Bail and Grealish, 2016), whereas under process accountability nurses must justify their decision-making processes to the head nurse in terms of the considerations that guided them in determining a specific treatment program or in prioritizing nursing care.

As for moderators, previous studies noted that the link between accountability focus and performance can be moderated by task complexity (Chang et al., 2013; Schulz-Hardt et al., 2020). Task complexity can range from low to high along three dimensions: component complexity, or the number of acts to be executed in the task performance; coordinative complexity, or the relationship between timing, frequency, intensity, and location requirements for task performance; and dynamic complexity, or individual adaptation to changes in the cause–effect chain during task performance (Wood, 1986). In nursing, for example, medication administration can be considered a simple or a complex task, depending on the three complexity dimensions (Smeulers et al., 2015).

Studies that examined the preferred combination of accountability focus and task complexity yielded equivocal answers, so it is not clear at this time which task is more appropriate for outcome vs. process accountability (Hall et al., 2017; Schulz-Hardt et al., 2020). Therefore, the main aim of this systematic review and meta-analysis was to investigate the strength of previous research evidence for performance under the outcome- vs. process-accountability focus and to understand whether the inconclusive findings could be attributed to moderating variables. Because nursing has a noticeably short history of empirical engagement with the concept, we deliberately broadened our review to include literature from the health sector as well as others. This is, to our knowledge, the first review of empirical literature on accountability undertaken to support nursing thinking, thereby contributing to the literature in several aspects. First, the synthesized evidence may enrich debates on nurses’ accountability by drawing on experiences from other sectors, and thus allowing for cross-pollination of ideas. Second, evidence on dimensions of accountability and moderating factors that might influence the accountability–performance link can contribute to the theoretical frameworks of accountability in nursing and inform the development of interventions to strengthen nurses’ quality of care.

## Materials and Methods

### Aim

The main aim of this systematic review and meta-analysis was to investigate the strength of previous research evidence for performance under the outcome- vs. process-accountability focus and to understand whether the inconclusive findings could be attributed to moderating variables.

### Design

A systematic review and meta-analysis of the literature following the Cochrane Handbook (Higgins et al., 2011) and the Preferred Reporting Items for Systematic Reviews and Meta-Analyses (PRISMA) guidelines was conducted (Page et al., 2021).

### Search Methods

A systematic search was conducted in five databases to examine the association between accountability focus (outcome vs. process) and task-performance outcomes: PsycINFO, Medline, PubMed, Scopus, and Cinahl. The search period was not limited by year of publication but ended in November 2020. Medical subject heading (MeSH) terms and search strings were used as follows: (“outcome accountability” AND [“Process accountability” OR “procedure accountability” OR “procedural accountability”]) in titles or abstracts. Duplicate references to the same records from multiple databases were removed. Additionally, we conducted manual searches of reference lists and searched for subsequent or prior studies published by the authors of the retrieved articles that could also meet our inclusion criteria.

**FIGURE 1:**
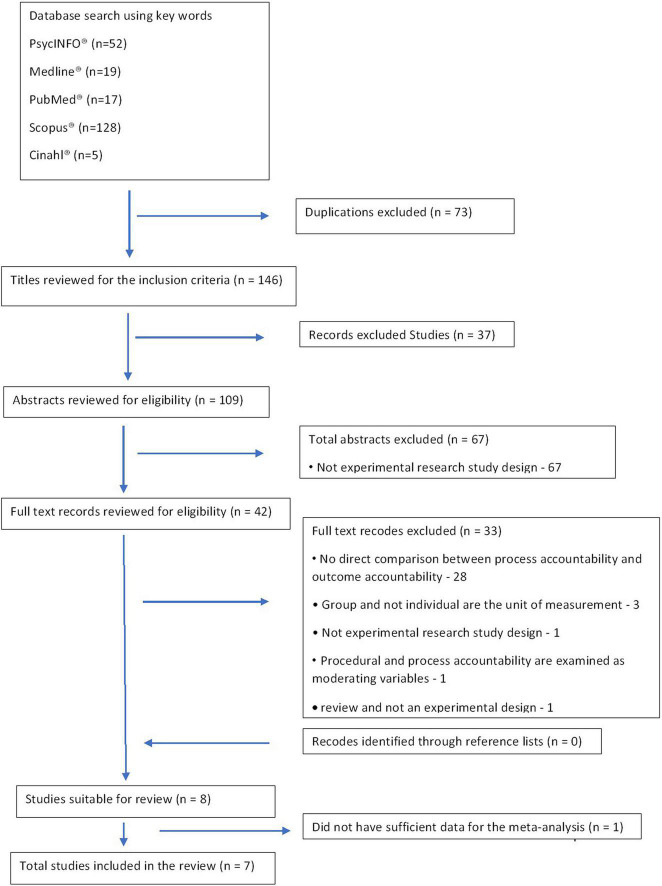
PRISMA flow diagram: study selection process.

Studies were included if they (a) were peer-reviewed journal articles (b) published in the English language, (c) employed an experimental design, and (d) compared the effects of outcome and process accountability on task performance. Studies were excluded if they did not refer to individual accountability. Figure 1 presents the PRISMA selection process.

### Search Outcomes

A total of 221 studies were retrieved and 73 duplicates were excluded (Figure 1). During the title, abstract, and full-text review process, 37, 67, and 33 studies, respectively, were excluded because they did not meet the inclusion criteria. Although eight studies met the criteria for inclusion in the final review, during the data-extraction process, one study (Siegel-Jacobs and Yates, 1996) lacked sufficient data for the meta-analysis (e.g., absence of means and SDs for task performance under outcome- and process-accountability focus). Attempts to contact the study’s authors failed; thus, that study was dropped from the meta-analysis.

### Quality Appraisal

Risk of bias assessment was assessed with the Revised Cochrane risk-of-bias tool for randomized trials (Higgins et al., 2011). Each study was tested and scored on a risk scale ranging from 1 (lowest risk) to 6 (highest risk) for each of the tool’s seven parameters: selection bias (random sequence generation and allocation concealment), reporting bias (selective reporting), other bias (other sources of bias), performance bias: blinding (participants and personnel), detection bias: blinding (outcome assessment), and attrition bias (incomplete outcome data). The risk score was calculated as the mean score across the seven items, indicating low (score 1 or 2), moderate (score 3 and 4), or high risk of bias (score 5 and 6).

Cohen’s kappa coefficient was calculated to determine the agreement between two independent reviewers regarding the consistency of the quality assessment of the included studies. Cohen’s kappa ranges from 0 to 1 and is interpreted as slight (≤0.20), fair (0.21–0.40), moderate (0.41–0.60), substantial (0.61–0.80), and almost perfect (≥0.81) agreement (Landis and Koch, 1977). Cohen’s kappa for this study was 0.90, indicating almost perfect agreement between raters.

In order to indicate possible publication bias, we used Doi plots and the LFK index; both are graphical methods for visualizing and quantifying asymmetry of studies, particularly when the number of studies is small (Furuya-Kanamori et al., 2018).

### Data Extraction

First, a systematic search of the five databases was conducted to retrieve titles and abstracts of potential literature, and thus identified and deleted duplicates. Second, titles were screened and excluded if they did not meet the inclusion criteria. Third, abstracts were read, and for those that met the inclusion criteria, the full text was also read (Figure 1).

### Task Complexity Assessment

Task complexity (low vs. high) was assessed by two independent reviewers, Ph.D. candidates who are experts in nursing, nurses’ accountability, and nursing tasks. The reviewers studied the experimental tasks employed and rated them on the three dimensions of task complexity—component, coordinative, and dynamic—using a Likert-type scale ranging from 0 (low task complexity) to 3 (high task complexity). Cohen’s kappa coefficient was 0.88, indicating almost perfect agreement between reviewers. Task complexity was determined to be low when the mean score of complexity was 0 or 1 and high when it was 2 or 3.

### Synthesis

Synthesis was conducted to assess the performance of individuals under different accountability focuses (outcome vs. process) across the included studies. All eligible studies were kept for meta-analysis regardless of their quality score, as relatively scarce research has been conducted in the field. Statistical analysis was performed using IBM SPSS version 25.0 (IBM, Armonk, New York). The pooled effect sizes (ESs) of studies and 95% confidence intervals (95%CIs) for the differences in performance means between outcome- and process-accountability focus were calculated using means and standard deviations (SDs) for each individual study. Cohen’s *d* and its corresponding 95%CI were calculated using those means and SDs. The ESs were interpreted as small (≤0.20), medium (0.20–0.80), or large (≥0.81) (Cohen, 1988). Because of the small number of studies included in the meta-analysis, the Q test could not be used to test for the variability in ES that is due to heterogeneity rather than to chance. Instead, we used the I^2^ (%) statistic, a function of the Q test (I^2^ = 100%*(Q-*df*)/Q) that does not depend on the number of included studies. The I^2^ statistic is interpreted as low (25%), moderate (50%), or high (75%) (Higgins et al., 2011). An inverse variance model was performed to estimate ESs in the pooled meta-analysis. The model was performed using MetaXL 5.3 to calculate the individual and pooled ESs (Barendregt and Doi, 2016).

## Results

### Characteristic of the Included Studies

Two studies (Davis et al., 2007; Patil et al., 2017) were inserted twice because they described two independent experiments. Thus, this meta-analysis includes seven studies representing nine experiments (Table 1). All seven studies were published between 2002 and 2017 and included a combined 1,080 participants, ranging from 30 to 422 per experiment. All studies provided direct comparison of task performance under process- and outcome-accountability focus. All studies employed experimental designs, manipulating process and outcome accountability focus. The manipulation directed the participants’ attention to how their performance will be judged – outcomes or process. To evaluate the participants’ perceptions of the process and outcome accountability focus the studies used similar manipulation checks. Among the nine experiments included in the systematic review, five were conducted in the United States (US; Brtek and Motowidlo, 2002; Davis et al., 2007; de Langhe et al., 2011; Chang et al., 2017; Patil et al., 2017), one was conducted in the US and Australia (Chang et al., 2013), and one was conducted in Germany (Häusser et al., 2017). Six experiments included undergraduate students; one (Chang et al., 2017) recruited professional participants from a variety of sources, such as research centers and science blogs. In addition, all experiments were conducted in a laboratory; no field experiments were observed.

**TABLE 1:** Characteristics of the included studies.

References	Country	No. participants: outcome; process	Type of task	Task performance testing
Chang et al., 2017	US	207; 215	Forecasting tournament	Participants’ forecast accuracy due to brier score
Patil et al., 2017 *	US	39; 40	Predicting performance of job applicants: on task requiring abstract thinking	Participants’ predictions were compared with ideal job applicant’s performance
Patil et al., 2017 *	US	39; 40	Predicting performance of job applicants: on task requiring orderly processes	Participants’ predictions were compared with ideal job applicant’s performance
Brtek and Motowidlo, 2002	US	80; 0	Validity of interview judgments	Validity of participants’ interviews judgments for predicting supervisors’ ratings of job performance
Häusser et al., 2017	Germany	37; 36	Idea generation: product development task	Number of ideas generated by participants
Chang et al., 2013	US + Australia	15; 15	Negotiating a sales contract	Sum of costs incurred within a participant’s negotiation
de Langhe et al., 2011	US	43; 44	Predicting popularity of easyphones	Measure of judgment quality, between participants’ predicted and real popularity scores for easyphones
Davis et al., 2007 *	US	44; 46	Computer-based decision-making simulation: first time	First-time participants’ performance on the computer-based decision-making simulation performance trial
Davis et al., 2007 *	US	207; 215	Computer-based decision-making simulation: second time	Second-time participants’ performance on the computer-based decision-making simulation performance trial

* Two different experiments were reported in this study; thus, both are addressed.

*US, United States.*

### Risk of Bias Assessment

**FIGURE 2:**
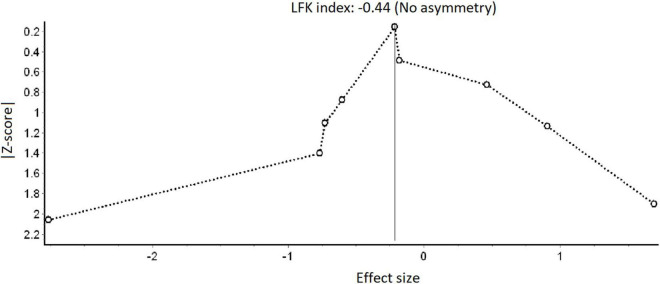
Doi plot: possible publication bias.

Overall, study quality across the nine experiments was moderate (mean score = 3). All had an adequate blinding for selection and reporting bias, and all but one (Chang et al., 2013) were randomized at the individual level. Only three experiments were assessed as adequate in detection bias sequence generation (Brtek and Motowidlo, 2002; Davis et al., 2007; Chang et al., 2017) and only two were assessed as adequate for attrition bias (Chang et al., 2013; Häusser et al., 2017). While in four experiments the risk for detection bias blinding was high (de Langhe et al., 2011; Chang et al., 2013; Häusser et al., 2017; Patil et al., 2017), in five experiments attrition bias was not well described (Brtek and Motowidlo, 2002; Davis et al., 2007; de Langhe et al., 2011; Chang et al., 2017; Patil et al., 2017). Figure 2 presents the Doi plot, which is used to give researchers an indication of whether there is any possible publication bias in the study. The Doi plot indicates that the included studies are quite symmetrical (LFK index = 0.44).

### The Effect of Accountability Focus on Task Performance

The overall pooled effect of the nine experiments on task performance yields no significant differences between outcome- and process-accountability focus, with a standardized mean difference of −0.09 (95%CI: −0.21, 0.03). However, this could have been a result of the high heterogeneity of the included experiments (I^2^ = 96%; chi-square *p*-value <0.001; Q = 180.01). Therefore, a moderating variable was considered.

### The Moderating Role of Task Complexity

**FIGURE 3:**
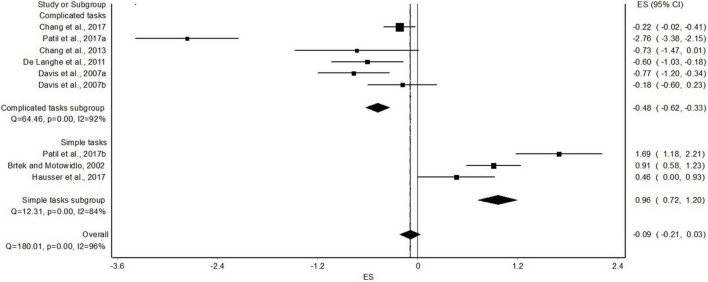
High-vs. low-complexity tasks: subgroup analysis.

Of the nine experiments, the tasks in six were characterized as high-complexity tasks, and in three experiments the tasks were characterized as low-complexity tasks (Table 2). A significant moderating role of task complexity was demonstrated (Figure 3). Specifically, whereas in the high-complexity-tasks subgroup, outcome accountability was associated with better task performance than process accountability (standardized mean difference of −0.48 [95%CI: −0.62, −0.33]), in the low-complexity-tasks subgroup, process accountability was associated with better task performance than outcome accountability (standardized mean difference of 0.96 [95% CI: 0.72, 1.20]). However, the heterogeneity was still high in both subgroups: I^2^ = 92% and I^2^ = 84% for the high- and low-complexity-task subgroups, respectively (*p* < 0.001).

**TABLE 2:** Score of task complexity.

References	Type of task	Dimension of task complexity			Total score	High/low complexity
		Component	Coordinative	Dynamic		
Chang et al., 2017	Forecasting tournament	1	1	1	3	High
Patil et al., 2017	Predicting performance of job applicants: on task requiring abstract thinking	0	1	1	2	High
Patil et al., 2017	Predicting performance of job applicants: on task requiring orderly processes	0	0	1	1	Low
Brtek and Motowidlo, 2002	Validity of interview judgments	0	1	0	1	Low
Häusser et al., 2017	Idea generation: product development task	0	0	0	0	Low
Chang et al., 2013	Negotiating a sales contract	0	1	1	2	High
de Langhe et al., 2011	Predicting popularity of easyphones	1	1	1	3	High
Davis et al., 2007	Computer-based decision-making simulation: first time	1	1	1	3	High
Davis et al., 2007	Computer-based decision-making simulation: second time	1	1	0	2	High
						

## Discussion

This meta-analysis addressed the dilemma of which accountability focus—process or outcome accountability—is superior for motivating better performance. This dilemma arises just as scholars and practitioners increasingly agree that, as committed as healthcare managers are to quality care, they paradoxically exert less control over the performance of bedside professionals than their manufacturing counterparts do (Schneider et al., 2009). Because of various healthcare restructuring efforts, these managers reported feeling distanced from the bedside and obliged to attend to more bureaucratic and administrative matters than to provide bedside care to patients and supervise staff behavior (Drach-Zahavy and Somech, 2013). Cultivating personal accountability may serve as a countermeasure for healthcare administrators to motivate employees to provide quality care for patients (Drach-Zahavy and Leonenko, 2019). Yet, apparently, the research on process vs. outcome accountability focus to date has raised more questions than answers, and whether it is more beneficial to emphasize outcome or process accountability remains relatively unsolved. This meta-analysis contributed to the dilemma in several aspects.

First, our findings provide, for the first time, accumulated empirical evidence regarding the nonsignificant direct superiority of neither accountability focus – process nor outcome. Namely, accountability focus by itself cannot serve as the sole motivator of better performance. This finding is especially crucial in nursing, where it has been typically assumed that a focus on outcomes is more important than a focus on processes (Monteiro Mantovani et al., 2017; Taghavi Larijani and Saatchi, 2019; Othman et al., 2020). For example, the Nursing Outcomes Classification (NOC), which was first developed in 1991 and which constitutes a comprehensive, standardized classification of patient outcomes, includes 540 nursing outcomes, without any reference to nursing process (Moorhead et al., 2018). However, as the meta-analysis findings indicated, in several instances, process accountability may outperform outcome accountability and lead to better performance.

Second, and closely related to the former point, the findings of the meta-analysis demonstrated that task complexity moderates the link between accountability focus and task performance, such that outcome accountability exerts a beneficial effect in more complex tasks whereas process accountability improves the performance of simpler tasks. This finding supports the resource allocation theory, suggesting that performing simple tasks requires fewer resources whereas performing complex tasks may exceed the performer’s resource capacity (Niessen and Lang, 2020). Given this resource-capacity limitation, simple tasks allow individuals under process (compared with outcome) accountability to focus on investing their resources wisely, being more attentive to the information they receive, and analyzing it adequately, which in turn yields optimal outcomes (Brtek and Motowidlo, 2002; Hall et al., 2017). However, by focusing the attention of the performer on the outcome of simpler tasks, outcome accountability encourages them to adhere to familiar cognitive patterns rather than to explore the task, attend to all information cues, and consider all task components. Thus, an outcome-accountability focus may preserve the performers’ resources, and achieve faster outcomes, at the price of suboptimal performance (Tetlock and Mellers, 2011; Patil et al., 2014). Indeed, in the nursing field, studies have shown that overload may lead nurses to be oriented toward outcomes and workarounds, leading to suboptimal performance (Hammoudi et al., 2018).

A mirror image emerges when performers engage with more complex tasks, which are intrinsically resource-intensive (Wood, 1986; Niessen and Lang, 2020). Coupled with the resource demands of process accountability (Brtek and Motowidlo, 2002), the combined circumstances of performing complex tasks under process accountability may become challenging and even tax the performer’s resource capacity. The performer might feel overwhelmed because of the urge to explore the task components, especially for tasks where attending to task components becomes challenging. Consequently, performance may decrease (Wood, 1986; Slaughter et al., 2006). In other words, complex tasks may require activation of many cognitive processes and/or management of considerable uncertainty, which most people cannot handle (Tetlock and Mellers, 2011; Patil and Tetlock, 2014). However, by focusing attention on the outcome of more complex tasks (i.e., outcome accountability), performers may stick to immediate outcomes, save their resources (Hall et al., 2017), and thus achieve improved performance (Tetlock and Mellers, 2011; Patil et al., 2014). This can be observed among nurses: in complex situations, such as medication administration or treating complex patients, nurses may need to focus on outcomes, thus saving psychological and physical resources and better performing their duties (Akter et al., 2018; Hammoudi et al., 2018).

### Limitations

These findings should be interpreted with caution, given the results of this meta-analysis, pertaining to the modest number of studies probing the issue so far, the moderate quality of the studies, the high heterogeneity among them, the fact that none included healthcare professionals, and that most were limited to a laboratory experimental study design with students mainly in the United States. Together, these limitations curb the conclusions that could be drawn from previous research (Shadish et al., 2002; Guo and Fraser, 2014). Nevertheless, there is no minimum number of studies for conducting a meta-analysis; that is, meta-analyses with a small number of studies can certainly provide reliable information about the questions they raise (Pigott, 2012). As Cohn and Becker (2003) stated, increasing the number of studies in meta-analysis does not necessarily increase the statistical power (Cohn and Becker, 2003).

Second, re-examination of the included studies revealed several additional potential moderators, including duration and type of training (Davis et al., 2007), participants’ characteristics (Chang et al., 2017), and task type (e.g., demanding creativity, quality, precision). However, the number of included studies examining each of these potential moderators (*n* = 1) did not allow us to test their effects in the meta-analysis.

Finally, one study (Siegel-Jacobs and Yates, 1996) failed to report information needed to calculate the effect size of its findings. Attempts to contact the authors failed; thus, this study was dropped from the meta-analysis. Exclusion of this study may have somewhat biased our findings.

### Implications for Practice and Research

The findings of the study have implications for both practice and research. The research findings suggesting outcome accountability is preferable in cases of more complex tasks carry important practical implications. Outcome accountability may be the type of accountability that nurses should act according to in places where it is required to make decisions in complex and uncertain situations, such as hospital and intensive care and emergency departments (Turale et al., 2020). This is especially relevant during this period, at the time of COVID-19 outbreak, as nurses work in wards caring for corona patients, which are characterized by uncertain, stressful, and complex tasks. On the other hand, nurses who work in the community, which is usually characterized by chronic patients and simpler and more routine tasks compared to the hospital, prefer to act in accordance with process accountability.

As for research, systematically understanding the impact of accountability-focus as well as its boundaries requires further research. First, Hall et al. (2017) identified six areas of moderating variables that might affect the accountability–performance link: personal characteristics of the performer, characteristics of the audience, task characteristics, context conditions, affective variables, and cultural variables. This meta-analysis focused on task characteristics. Further studies should explore other possible moderators explaining the accountability focus–performance link. For example, in line with the performance-resource function (Niessen and Lang, 2020), a training period enabling participants to become acquainted with a task may decrease the resources needed to perform the task. Unfortunately, only one study reported training before task performance (Davis et al., 2007); thus, we could not test the moderating effects of training. Further, only a few studies investigated the moderating role of personality traits and found that these mattered for performing tasks under different accountability focuses, such as goal orientation (Davis et al., 2007). Future studies should explore other personal attributes such as the Big 5 personality traits (Hall et al., 2017; Royle, 2017) or personal decision-making styles (Lepri et al., 2018) as moderators in the accountability focus–performance link.

Second, because of the moderate quality of current experiments, future studies should consider conducting experiments with higher methodological quality that reduces biases: for example, using a double-blind randomized assignment design where both participants and assessors are blinded to the group assignment (Shadish et al., 2002).

Third, previous studies employed experimental designs, thereby constraining the understanding of how accountability focus unfolds in real life, and particularly in the routine work of nurses. Field studies that derive information about how nurses perceive the accountability focuses on their ward are warranted to gain external support to the current understanding this issue in the literature. Furthermore, there is a need for additional research that will give head nurses in inpatient wards tools for motivating nursing staff (namely accountability focus) to their best performance, on tasks with different levels of complexity.

Finally, the global COVID-19 pandemic raises questions regarding the role of nurses’ accountability during pandemic. Accordingly, studies should investigate the impact of nurses’ accountability in general, as well as outcome- vs. process-accountability focus, during pandemic times, and compare the results with those before pandemics. Specifically, do crises, such as the pandemic outbreak, encourage implementation of outcome- or process-focused accountability, and how does this increase or precisely relieve nurses’ levels of strain and quality of care?

## Conclusion

This meta-analysis sheds light on the dilemma about which accountability focus, process accountability or outcome accountability, is superior for motivating better performance. Accountability focus alone cannot be the sole motivator for improving performance, since task complexity moderates the relationship between accountability focus and performance. Outcome accountability exerts a beneficial effect in complex tasks, whereas process accountability improves performance in simple tasks. The findings are crucial in nursing, where it has long been assumed that the emphasis should be on outcomes rather than processes. However, no study to date has focused on nurses; thus, there is a crucial need for high-quality experiments with this population.

## Data Availability Statement

The original contributions presented in the study are included in the article/supplementary material, further inquiries can be directed to the corresponding author.

## Author Contributions

IS, AD-Z, and ES: study design, data collection, data analysis, study supervision, manuscript writing, and critical revisions for important intellectual content. All authors meet the criteria for authorship have approved the final article and all those entitled to authorship are listed as authors and took part in conceptualization, methodology, software, validation, formal analysis, investigation, resources, data curation, writing – original draft, writing – review and editing, visualization, and supervision.

## Conflict of Interest

The authors declare that the research was conducted in the absence of any commercial or financial relationships that could be construed as a potential conflict of interest.

## Publisher’s Note

All claims expressed in this article are solely those of the authors and do not necessarily represent those of their affiliated organizations, or those of the publisher, the editors and the reviewers. Any product that may be evaluated in this article, or claim that may be made by its manufacturer, is not guaranteed or endorsed by the publisher.

## References

[B1] Akter N. Akkadechanunt T. Chontawan R. Klunklin A. (2018). Factors predicting quality of work life among nurses in tertiary-level hospitals, Bangladesh. *Int. Nurs. Rev.* 65 182–189. 10.1111/inr.12401 29105085

[B2] Bail K. Grealish L. (2016). ‘Failure to Maintain’: a theoretical proposition for a new quality indicator of nurse care rationing for complex older people in hospital. *Int. J. Nurs. Stud.* 63 146–161. 10.1016/j.ijnurstu.2016.08.001 27658271

[B3] Barendregt J. J. Doi S. A. (2016). *MetaXL user guide, Version 4 2011–2016.* Queensland, Australia: EpiGear.

[B4] Brtek M. D. Motowidlo S. J. (2002). Effects of procedure and outcome accountability on interview validity. *J. Appl. Psychol.* 87 185–191. 10.1037/0021-9010.87.1.185 11916212

[B5] Buheji M. Buhaid N. (2020). Nursing human factor during COVID-19 pandemic. *Int. J. Nurs. Sci.* 10 12–24. 10.5923/j.nursing.20201001.02 22499009

[B6] Chang L. J. Cheng M. M. Trotman K. T. (2013). The effect of outcome and process accountability on customer-supplier negotiations. *Account. Organ. Soc.* 38 93–107. 10.1016/j.aos.2012.12.002

[B7] Chang W. Atanasov P. Patil S. Mellers B. A. Tetlock P. E. (2017). Accountability and adaptive performance under uncertainty: a long-term view. *Judgment Decision Making* 12 610–626.

[B8] Charania N. A. M. A. Ferguson D. L. Bay E. Freeland B. S. Bradshaw K. Harden K. (2017). A professionalism and safety code of conduct designed for undergraduate nursing students. *J. Prof. Nurs.* 33 460–463. 10.1016/j.profnurs.2017.06.006 29157576

[B9] Chen S.-L. Sun J.-L. Jao J.-Y. (2020). A predictive model of student nursing competency in clinical practicum: a structural equation modelling approach. *Nurse Educ. Today* 95:104579. 10.1016/j.nedt.2020.104579 33059277

[B10] Cohen J. (1988). *Statistical Power Analysis for the Behavioral Sciences*, 2nd Edn. Hillsdale, NJ: Erlbaum.

[B11] Cohn L. D. Becker B. J. (2003). How meta-analysis increases statistical power. *Psychol. Methods* 8 243–253. 14596489 10.1037/1082-989X.8.3.243

[B12] Combrinck Y. Van Wyk N. C. Mogale R. S. (2021). Preserving nurses’ professional dignity: six evidence-based strategies. *Int. Nurs. Rev.* 69 106–113. 10.1111/inr.12701 34292605

[B13] Davis W. D. Mero N. Goodman J. M. (2007). The interactive effects of goal orientation and accountability on task performance. *Hum. Perform.* 20 1–21. 10.1207/s15327043hup2001_1

[B14] De Dreu C. K. W. Beersma B. Stroebe K. Euwema M. C. (2006). Motivated information processing, strategic choice, and the quality of negotiated agreement. *J. Person. Soc. Psychol.* 90 927–943. 10.1037/0022-3514.90.6.927 16784343

[B15] de Langhe B. van Osselaer S. M. J. Wierenga B. (2011). The effects of process and outcome accountability on judgment process and performance. *Organ. Behav. Hum. Decision Proc.* 115 238–252. 10.1016/j.obhdp.2011.02.003

[B16] Drach-Zahavy A. Leonenko M. (2019). An accountability account? The diverse outcomes of perceived personal and team accountability. *Acad. Manage. Proc.* 2019:16384. 10.5465/AMBPP.2019.222

[B17] Drach-Zahavy A. Leonenko M. Srulovici E. (2018). Towards a measure of accountability in nursing: a three-stage validation study. *J. Adv. Nurs.* 74 2450–2464. 10.1111/jan.13735 29869349

[B18] Drach-Zahavy A. Somech A. (2013). Linking task and goal interdependence to quality service. *J. Service Manage.* 24 151–169. 10.1108/09564231311323944

[B19] Drach-Zahavy A. Srulovici E. (2019). The personality profile of the accountable nurse and missed nursing care. *J. Adv. Nurs.* 75 368–379. 10.1111/jan.13849 30209825

[B20] Furuya-Kanamori L. Barendregt J. J. Doi S. A. R. (2018). A new improved graphical and quantitative method for detecting bias in meta-analysis. *Int. J. Evid. Based Health.* 16 195–203. 10.1097/XEB.0000000000000141 29621038

[B21] Guo S. Fraser M. W. (2014). *Propensity Score Analysis: Statistical Methods and Applications.* Los Angeles, CA: SAGE.

[B22] Hall A. T. Frink D. D. Buckley M. R. (2017). An accountability account: a review and synthesis of the theoretical and empirical research on felt accountability. *J. Organ. Behav.* 38 204–224. 10.1002/job.2052

[B23] Hammoudi B. M. Ismaile S. Abu Yahya O. (2018). Factors associated with medication administration errors and why nurses fail to report them. *Scand. J. Caring Sci.* 32 1038–1046. 10.1111/scs.12546 29168211

[B24] Häusser J. A. Frisch J. U. Wanzel S. Schulz-Hardt S. (2017). Effects of process and outcome accountability on idea generation. *Exp. Psychol.* 64 262–272. 10.1027/1618-3169/a000368 28922995

[B25] Higgins J. P. T. Altman D. G. Sterne J. A. C. (2011). “Assessing risk of bias in included studies,” in *Cochrane Handbook for Systematic Reviews of Interventions*, Version 5.1.0 (updated March 2011), eds Higgins J. P. T. Green S. (London: Cochrane Collaboration)

[B26] Jackson D. Bradbury-Jones C. Baptiste D. Gelling L. Morin K. H. Neville S. (2020). International Nurses Day 2020: remembering nurses who have died in the COVID-19 pandemic. *J. Clin. Nurs.* 29 2050–2052. 32390287 10.1111/jocn.15315 PMC7272897

[B27] Landis J. R. Koch G. G. (1977). The measurement of observer agreement for categorical data. *Biometrics* 33 159–174. 10.2307/2529310 843571

[B28] Leonenko M. Drach-Zahavy A. (2016). “You are either out on the court, or sitting on the bench”: understanding accountability from the perspectives of nurses and nursing managers. *J. Adv. Nurs.* 72 2718–2727. 10.1111/jan.13047 27322101

[B29] Lepri B. Oliver N. Letouzé E. Pentland A. Vinck P. (2018). Fair, transparent, and accountable algorithmic decision-making processes. *Philos. Technol.* 31 611–627. 10.1007/s13347-017-0279-x

[B30] Li Y. Luo B. (2020). Frontline health-care workers in combating the COVID-19: respect and reflect. *Risk Manage. Health. Policy* 13 1119–1122. 10.2147/RMHP.S254639 PMC7440837 32884373

[B31] Mansouri M. Rowney J. I. A. (2014). The dilemma of accountability for professionals: a challenge for mainstream management theories. *J. Bus. Ethics* 123 45–56. 10.1007/s10551-013-1788-x

[B32] Monteiro Mantovani V. Rodríguez A. L. Lucena A. de Abreu M. Paz da Silva E. (2017). Nursing outcomes for the evaluation of patients during smoking cessation. *Int. J. Nurs. Know.* 28 204–210. 10.1111/2047-3095.12138 27247247

[B33] Moorhead S. Johnson M. Maas M. L. Swanson E. (2018). *Nursing Outcomes Classification (NOC)-e-book: Measurement of health outcomes.* St. Louis, MO: Elsevier.

[B34] Niessen C. Lang J. W. (2020). Cognitive control strategies and adaptive performance in a complex work task. *J. Appl. Psychol.* 106. 10.1037/apl0000830 [Epub online ahead of print] 33030921

[B35] Othman E. H. Shatnawi F. Alrajabi O. Alshraideh J. A. (2020). Reporting nursing interventions classification and nursing outcomes classification in nursing research: a systematic review. *Int. J. Nurs. Know* 31 19–36. 10.1111/2047-3095.12265 31743604

[B36] Page M. J. McKenzie J. E. Bossuyt P. M. Boutron I. Hoffmann T. C. Mulrow C. D. (2021). The PRISMA 2020 statement: an updated guideline for reporting systematic reviews. *BMJ* 372:n71. 10.1136/bmj.n71 33782057 PMC8005924

[B37] Patil S. V. Tetlock P. E. (2014). Punctuated incongruity: a new approach to managing trade-offs between conformity and deviation. *Res. Organ. Behav.* 34 155–171. 10.1016/j.riob.2014.08.002

[B38] Patil S. V. Tetlock P. E. Mellers B. A. (2017). Accountability systems and group norms: balancing the risks of mindless conformity and reckless deviation. *J. Behav. Decision Making* 30 282–303. 10.1002/bdm.1933

[B39] Patil S. V. Vieider F. Tetlock P. E. (2014). “Process versus outcome accountability,” in *The Oxford Handbook of Public Accountability*, eds Bovens M. Goodin R. E. Schillemans T. (Oxford, UK: Oxford University Press), 69–89.

[B40] Pigott T. (2012). *Advances in Meta-Analysis.* New York, NY: Springer.

[B41] Royle M. T. (2017). The mediating effect of felt accountability on the relationship between personality and job satisfaction. *Int. J. Manage. Market. Res.* 10 19–44.

[B42] Schneider B. Macey W. H. Lee W. C. Young S. A. (2009). Organizational service climate drivers of the American Customer Satisfaction Index (ACSI) and financial and market performance. *J. Serv. Res.* 12, 3–14. 10.1177/1094670509336743

[B43] Scholten L. van Knippenberg D. Nijstad B. A. De Dreu C. K. W. (2007). Motivated information processing and group decision-making: effects of process accountability on information processing and decision quality. *J. Exp. Soc. Psychol.* 43 539–552. 10.1016/j.jesp.2006.05.010

[B44] Schulz-Hardt S. Rollwage J. Wanzel S. K. Frisch J. U. Häusser J. A. (2020). Effects of process and outcome accountability on escalating commitment: a two-study replication. *J. Exp. Psychol.* 2020 112–124. 10.1037/xap0000321 32658527

[B45] Shadish W. R. Cook T. D. Campbell D. T. (2002). *Experimental and Quasi-Experimental Designs for Generalized Causal Inference.* Boston, MA: Houghton Mifflin.

[B46] Siegel-Jacobs K. Yates J. F. (1996). Effects of procedural and outcome accountability on judgment quality. *Organ. Behav. Hum. Decision Proc.* 65 1–17. 10.1006/obhd.1996.0001

[B47] Slaughter J. E. Bagger J. Li A. (2006). Context effects on group-based employee selection decisions. *Organ. Behav. Hum. Decision Proc.* 100 47–59. 10.1016/j.obhdp.2006.01.003

[B48] Smeulers M. Verweij L. Maaskant J. M. de Boer M. Krediet C. T. P. van Dijkum E. J. M. N. (2015). Quality indicators for safe medication preparation and administration: a systematic review. *PLoS One* 10:e0122695. 10.1371/journal.pone.0122695 25884623 PMC4401721

[B49] Srulovici E. Drach-Zahavy A. (2017). Nurses’ personal and ward accountability and missed nursing care: a cross-sectional study. *Int. J. Nurs. Stud.* 75 163–171. 10.1016/j.ijnurstu.2017.08.003 28829974

[B50] Stievano A. Tschudin V. (2019). The ICN code of ethics for nurses: a time for revision. *Int. Nurs. Rev.* 66 154–156. 10.1111/inr.12525 31124137

[B51] Taghavi Larijani T. Saatchi B. (2019). Training of NANDA-I Nursing Diagnoses (NDs), Nursing Interventions Classification (NIC) and Nursing Outcomes Classification (NOC), in psychiatric wards: a randomized controlled trial. *Nurs. Open* 6 612–619. 10.1002/nop2.244 30918711 PMC6419136

[B52] Tetlock P. E. Mellers B. A. (2011). “Structuring accountability systems in organizations: Key trade-offs and critical unknowns,” in *Intelligence analysis: Behavioral and social scientific foundations*, eds Fischhoff B. Chauvin C. (Washington, DC: National Academies Press), 249–270.

[B53] Turale S. Meechamnan C. Kunaviktikul W. (2020). Challenging times: ethics, nursing and the COVID-19 pandemic. *Int. Nurs. Rev.* 67 164–167. 10.1111/inr.12598 32578249 PMC7361611

[B54] Wood R. E. (1986). Task complexity: definition of the construct. *Organ. Behav. Hum. Decision Proc.* 37 60–82. 10.1016/0749-5978(86)90044-0

